# Risk assessment of antibody-mediated damage based on the detection of HLA and non-HLA antibodies toward extracellular antigens before kidney transplantation

**DOI:** 10.3389/fimmu.2025.1614408

**Published:** 2025-08-12

**Authors:** Eulàlia Solà-Porta, Dolores Redondo-Pachón, Jorge Eguía-Núñez, Anna Buxeda, José Luís Caro, Javier Gimeno, Luís Campuzano, Carla Burballa, Betty Chamoun, Sara Sanz-Ureña, Judith Federico-Vega, Elisenda Alari-Pahissa, Julio Pascual, María José Pérez-Sáez, Marta Crespo

**Affiliations:** ^1^ Nephropathy Research Group, Hospital del Mar Medical Research Institute, Barcelona, Spain; ^2^ Department of Nephrology, Hospital del Mar, Barcelona, Spain; ^3^ Immunology Department, Laboratori de Referència de Catalunya SA, El Prat de Llobregat, Spain; ^4^ Immunology Department, Hospital Clinic, Barcelona, Spain; ^5^ Department of Pathology, Hospital del Mar, Barcelona, Spain; ^6^ Department of Nephrology, Hospital Universitario 12 de Octubre, Madrid, Spain

**Keywords:** non-HLA antibodies, HLA, ABMR, antibody-mediated rejection, MVI, microvascular inflammation, GSTT1

## Abstract

**Introduction:**

Donor-specific human leukocyte antigens antibodies (HLA-DSA) contribute toantibody-mediated rejection (ABMR) after kidney transplantation (KT). Non-HLA antibodies may play a role in ABMR in the presence of HLA-DSA or the development of microvascular inflammation (MVI) in its absence. Considering both types of antibodies in potential recipients could enhance ABMR/MVI risk assessment.

**Methods:**

We present a case-control study of 121 KT recipients, 46 with ABMR/ MVI diagnosis, and 75 control cases with available sera before and after KT, follow-up HLA antibody monitoring, and biopsies. We determined 60 serum non-HLA antibodies using a multiplex test with an established cutoff. We evaluated their association with ABMR/MVI using a sample median fluorescence intensity (MFI) ratio sum.

**Results:**

Following commercial cutoffs, non-HLA antibodies were detected in 87% of the patients before KT. We found that a high non-HLA antibody MFI ratio sum before KT and at biopsy were associated with an increased risk of ABMR/MVI, independently of HLA sensitization or HLA-DSA (OR = 1.039, p = 0.014 and OR = 1.036, p = 0.024). Antibodies against extracellular non-HLA antigens were associated with ABMR/MVI before KT (OR = 1.053, p = 0.040), but at diagnosis, only antibodies against intracellular non-HLA antigens were associated (OR = 1.062, p = 0.018).

**Conclusion:**

These findings suggest that non-HLA antibody assessment offers valuable complementary information, regardless of HLA sensitization, though appropriate cut-offs should be explored.

## Introduction

In kidney transplantation (KT), antibody-mediated rejection (ABMR) remains a barrier to long-term graft survival (1, 2). Donor-specific antibodies (DSA) against human leukocyte antigens (HLA) are central to the pathogenesis of ABMR by triggering endothelial damage and complement activation, innate and adaptive immune responses, and intracellular pathways (3–7). The presence of HLA-DSA, both before and after transplantation, has been strongly associated with ABMR and graft loss (8–10).

The 2017 and 2019 Banff classifications defined ABMR based on compatible histology and histological signs of interaction between the endothelia and antibodies—such as microvascular inflammation (MVI)—in the presence of HLA-DSA or C4d deposits in peritubular capillaries (11, 12). However, ABMR damage can occur even in the absence of detectable HLA-DSA, with antibody involvement inferred from complement activation (13). A subset of patients presents with isolated MVI in the absence of HLA-DSA or complement activation. Although not categorized in these Banff classifications, this pattern of damage influences graft survival (7, 14, 15) and shares certain injury mechanisms with AMBR (7, 16), although not all. The most recent Banff 2022 classification (17) now includes these patients under Category 2, as a subcategory termed *MVI, DSA-negative, and C4d-negative*.

There has been a growing interest in the potential role of antibodies directed against non-HLA molecules in ABMR (18). Initial studies employed tests that allowed the evaluation of reactions against specific targets such as the major histocompatibility complex class I-related chain A (MICA), angiotensin receptor 1, and endothelin receptor, among others. Although these antibodies have been variably associated with graft outcomes, none of them has shown as strong a correlation with ABMR as HLA-DSA (19–21).

The damage mechanisms of non-HLA antibodies appear to involve a mixture of alloimmune and autoimmune responses depending on each antigen (22). Some antigens induce an alloimmune response based on a non-HLA genetic mismatch between donor and recipient, such as antibodies against MICA or anti-glutathione S-transferase theta 1 (GSTT1) (23). In contrast, most non-HLA antigens seem to induce an autoimmune reaction, as shown in the case of antibodies against self-molecules like angiotensin type 1 receptor, endothelin receptor, or rho GDP dissociation inhibitor beta (24). This raises important questions about why these antibodies target the kidney graft specifically, but not the native kidneys or other organs (25).

The growing identification of non-HLA targets has driven the development of novel and user-friendly detection tests leveraging versatile technology. Multiplex bead panels and antibody detection immunoassays now enable simultaneous testing of multiple non-HLA targets. Using these tools, recent studies have focused on the overall non-HLA antibody burden and its impact on ABMR and graft survival (26–30). Several unresolved questions remain (31), such as which non-HLA antibodies should be included in a multiplex panel, and at what stage in the transplant process should be tested.

In this study, we aimed to characterize a set of non-HLA antibodies in KT recipients with ABMR/MVI and compare them to a control group. Our objective was to evaluate their potential association with ABMR/MVI development paralleling current immune risk assessment strategies based on the patient’s HLA antibody sensitization while on the waiting list.

## Materials and methods

### Study design and study population

We conducted an observational case-control study that included KT recipients who underwent KT at Hospital del Mar, Barcelona, Spain. The case group included 46 patients who were transplanted between 2006 and 2019, diagnosed with ABMR/MVI (23 with detectable HLA-DSA at diagnosis and 23 without), and with available follow-up protocol or indication biopsies and serum samples both before KT and at the time of the biopsies. The control group consisted of 75 patients selected from a cohort of 131 consecutive KT recipients who were transplanted between 2013 and 2018 at the same center. Eligible controls had available protocol or indication biopsies at 1 and 3 years after KT and serum samples collected before KT and at the time of each biopsy. Patients with damage (Categories 2, 3, 4, and MVI of the Banff 2017 classification) in one or both biopsies were excluded.

**Figure 1:**
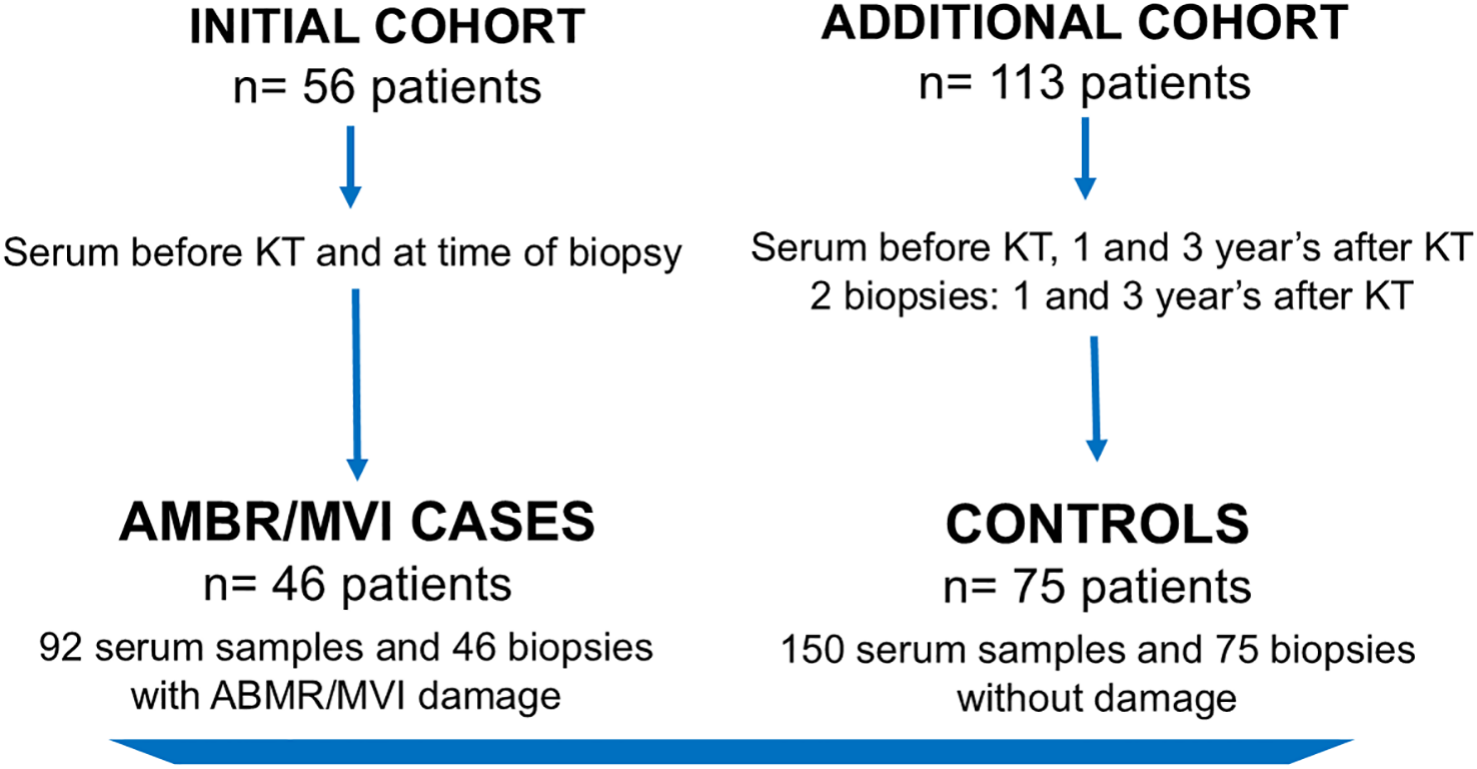
Population flow chart.

The final study population included 121 patients: 46 cases with ABMR/MVI and 75 control cases without damage (**Figure 1**). For the present analysis, only sera and biopsies 1 year after KT were considered from control cases, knowing that later biopsies did not show ABMR/MVI either. Patients’ serum samples were tested for HLA and non-HLA antibodies before KT and at the time of biopsy.

### HLA antibody detection technique

HLA antibody analysis was performed as previously described (9). In brief, serum samples were first evaluated with screening kits using Lifecodes LifeScreen Deluxe assay (Immucor GTI Diagnostics, Inc^®^). Depending on the screening positivity for class I or II, further analysis was performed using Lifecodes LSA Class I and/or Class II test (Immucor GTI Diagnostics, Inc^®^), according to the manufacturer’s instructions. Finally, samples were analyzed on a Luminex 200 (Bio-Rad Luminex^®^) using Bio-Plex Manager 6.2 for data acquisition and Match It! Antibody v.1.1.0.2 for analysis. The bead was considered positive when at least two of the following values were exceeded: background corrected median fluorescence intensity calculated for each bead >1,000, background corrected ratio >5, and antigen density background corrected ratio >5 under review by two researchers (DRP and JE).

### Non-HLA antibody detection technique

Non-HLA antibodies in serum were analyzed with a Lifecodes multiplex bead panel by Immucor^®^ GTI Diagnostics, Inc. (lot num. 3010187) comprising 60 non-HLA antigens conjugated to polystyrene beads (**Supplementary Table 1**). Forty microliters of non-HLA beads and 10 µl of patient serum were added to each well and then incubated on a rotating platform for 30 min in the dark at room temperature. After incubation, wells were washed to remove unbound antibodies, and 50 µl of a previously diluted phycoerythrin-conjugated anti-human IgG (5 µl of conjugate diluted with 45 µl of wash buffer) was added and further incubated for 30 min in the dark on a rotating platform at room temperature. Samples were analyzed on a Luminex 200 (Bio-Rad Luminex^®^), and a median fluorescence intensity (MFI) was obtained for each bead.

According to manufacturer instructions, a background corrected MFI and MFI ratio with the cutoff value defined by the manufacturer (MFI/cutoff value) was calculated for each target antigen. Non-HLA antibodies were considered positive when the background corrected MFI value was above the given cutoff, and the MFI ratio was equal to or above 1. For each sample, an absolute count of positive non-HLA antibodies was calculated, as well as an intensity count based on the total sum of the MFI ratio values (MFI/cutoff value) of the positive non-HLA antibodies.

The 60 non-HLA antibodies included in the panel and the cutoffs given by the manufacturer are listed in **Supplementary Table 1**.

### Biopsies

All biopsies were reviewed and retrospectively scored by one pathologist using the Banff 2017 classification and allocated to any of the six Banff diagnostic categories in agreement with researchers (JG, DRP, and AB). Biopsies were categorized as ABMR/MVI for this study when they fulfilled the biopsy diagnosis of ABMR (Banff category 2) or had MVI (glomerulitis and/or peritubular capillaritis ≥2) in the absence of HLA-DSA.

### Variables

Clinical data were collected from our local transplant database, including baseline demographic characteristics, transplant characteristics, and periodically registered clinical follow-up variables. For immunological sensitization evaluation before KT, a calculated panel of reactive antibodies (cPRA), using HLA frequencies derived from local bone marrow donors active in Catalunya, was used for the study and clinical KT prioritizing programs. Patients were followed until graft loss (return to dialysis or retransplantation), death, and end of follow-up (1 July 2022).

### Statistical analysis

Based on the data distribution, the results of quantitative variables are expressed as mean and standard deviation (SD) or as median and interquartile range (IQR). Categorical variables are described as counts and percentages. Comparisons between groups were done using the Student’s t-test for parametric continuous variables and the U Mann–Whitney test for non-parametric. For categorical variables, a chi-square test was used. Univariable and multivariable logistic regression analyses were used to evaluate odds ratios (ORs) for the occurrence of ABMR/MVI according to the MFI ratio sum and absolute number of non-HLA antibodies and for the individual non-HLA antibodies. Results are expressed as OR and 95% confidence interval (CI). A p-value <0.05 was considered statistically significant, while a p-value <0.1 was considered significant to be included in the multivariable analysis. Statistical analysis was performed using SPSS V 20 (SPSS Inc., Chicago, IL, USA).

## Results

### Population characteristics

The baseline demographics and clinical characteristics of the 121 patients included are presented in **Table 1**. A total number of 95 (78.5%) patients received a deceased kidney allograft, 15 (12.4%) underwent preemptive transplantation, and 11 (9.1%) received a repeated KT. Comparison between cases and controls, defined by the presence or absence of ABMR/MVI, showed no significant differences in recipient or donor demographics, except for a higher proportion of female recipients in the ABMR/MVI group (60.9% vs. 34.7%, p = 0.005). Regarding transplantation characteristics, we found, as expected, that patients in the ABMR/MVI group had a higher frequency of pretransplant HLA antibodies (cPRA over 50%, 28.3% vs. 9.3%, p = 0.007) and preformed HLA-DSA (19.6% vs. 2.7%, p = 0.002).

**Table 1:** Baseline characteristics.

	All patients (n = 121)	ABMR/MVI (n = 46)	Control group (n = 75)	p-Value
Recipient demographics				
Sex (female), n (%) Age (years), mean ± SD Ethnicity (Caucasian), n (%) Body mass index (kg/m^2^), mean ± SD Type 2 diabetes, n (%) Cause of end-stage renal, n (%) - Immune-mediated disease - Cystic kidney disease - Diabetic nephropathy - Vascular disease - Tubulointerstitial disease - Unknown - Other disease KT preemptive, n (%) Median time on dialysis (months, IQR)	54 (44.6) 54.5 ± 13.2 99 (81.8) 27.0 ± 5.7 36 (29.8) 22 (18.2) 15 (12.4) 19 (15.7) 15 (12.4) 11 (9.1) 29 (24) 10 (8.3) 15 (12.4) 20 (9–34.5)	28 (60.9) 53.7 ± 14.6 39 (84.8) 27.1 ± 6.3 13 (28.3) 8 (17.4) 7 (15.2) 7 (15.2) 5 (10.9) 2 (4.3) 13 (28.3) 4 (8.6) 7 (15.2) 16 (11–41)	26 (34.7) 54.9 ± 12.3 60 (80.0) 27.0 ± 5.3 23 (30.7) 14 (16.5) 10 (11.8) 13 (15.2) 11 (12.9) 10 (11.8) 21 (24.7) 6 (7.1) 8 (10.7) 20.5 (9–33.8)	0.005 0.637 0.508 0.875 0.779 0.440 0.461 0.775
Donor demographics				
Sex (female), n (%) Age (years), mean ± SD Donor type, n (%) - Living donor - Donation after brain death - Donation after cardiac death Median cold ischemia time (hours, IQR) Delayed graft function, n (%)	58 (48.7) 55.7 ± 14.4 26 (21.5) 70 (57.9) 25 (20.7) 12 (5–16.5) 31 (25.6)	20 (45.5) 54.8 ± 15.4 10 (21.7) 28 (60.9) 8 (17.4) 13 (7–16) 15 (32.6)	38 (50.7) 56.3 ± 13.7 16 (21.3) 42 (56) 17 (22.7) 10.5 (4.6–17) 16 (21.3)	0.583 0.569 0.778 0.530 0.168
Transplant characteristics				
HLA cPRA over 50% before KT HLA-DSA before KT, n (%) - HLA-DSA class II Previous transplantation, n (%) Previous pregnancies, n (%) (n = 59) Blood transfusion before KT, n (%) Induction therapy (thymoglobulin), n (%) Immunosuppression regimen (tacrolimus, mycophenolate, and steroids), n (%)	20 (16.5) 11 (9.1) 8 (72.7) 11 (9.1) 44 (81.5) 47 (38.8) 18 (15) 95 (78.5)	13 (28.3) 9 (19.6) 6 (66.7) 7 (15.2) 23 (82.1) 20 (43.5) 11 (24.4) 35 (76.1)	7 (9.3) 2 (2.7) 2 (100) 4 (5.3) 21 (80.8) 27 (36) 7 (9.3) 60 (80)	0.007 0.002 0.066 0.897 0.413 0.025 0.611
Follow up				
Median time of follow-up (months, IQR) Graft loss death censored at end of follow-up, n (%)	79 (58–98) 15 (12.4)	77 (51–106) 12 (26.1)	81.5 (64–95) 3 (4)	0.309 <0.001
Graft function at biopsy				
Median time from KT to biopsy (months, IQR) Renal function: - Serum creatinine (mg/dl) mean ± SD - eGFR (MDRD) (ml/min) mean ± SD - Urine protein/creatinine ratio (mg/g) mean ± SD HLA-DSA, n (%)	13 (12–16) 1.6 ± 0.7 49.3 ± 20.3 336 ± 462.4 26 (21.5)	14 (12–37.3) 1.8 ± 0.9 44.7 ± 17.4 428.9 ± 483.2 23 (50)	13 (12–15) 1.5 ± 0.6 52 ± 21.6 283.2 ± 450 3 (4)	0.016 0.050 0.070 0.084 <0.001

### Non-HLA antibodies before transplantation

**Figure 2:**
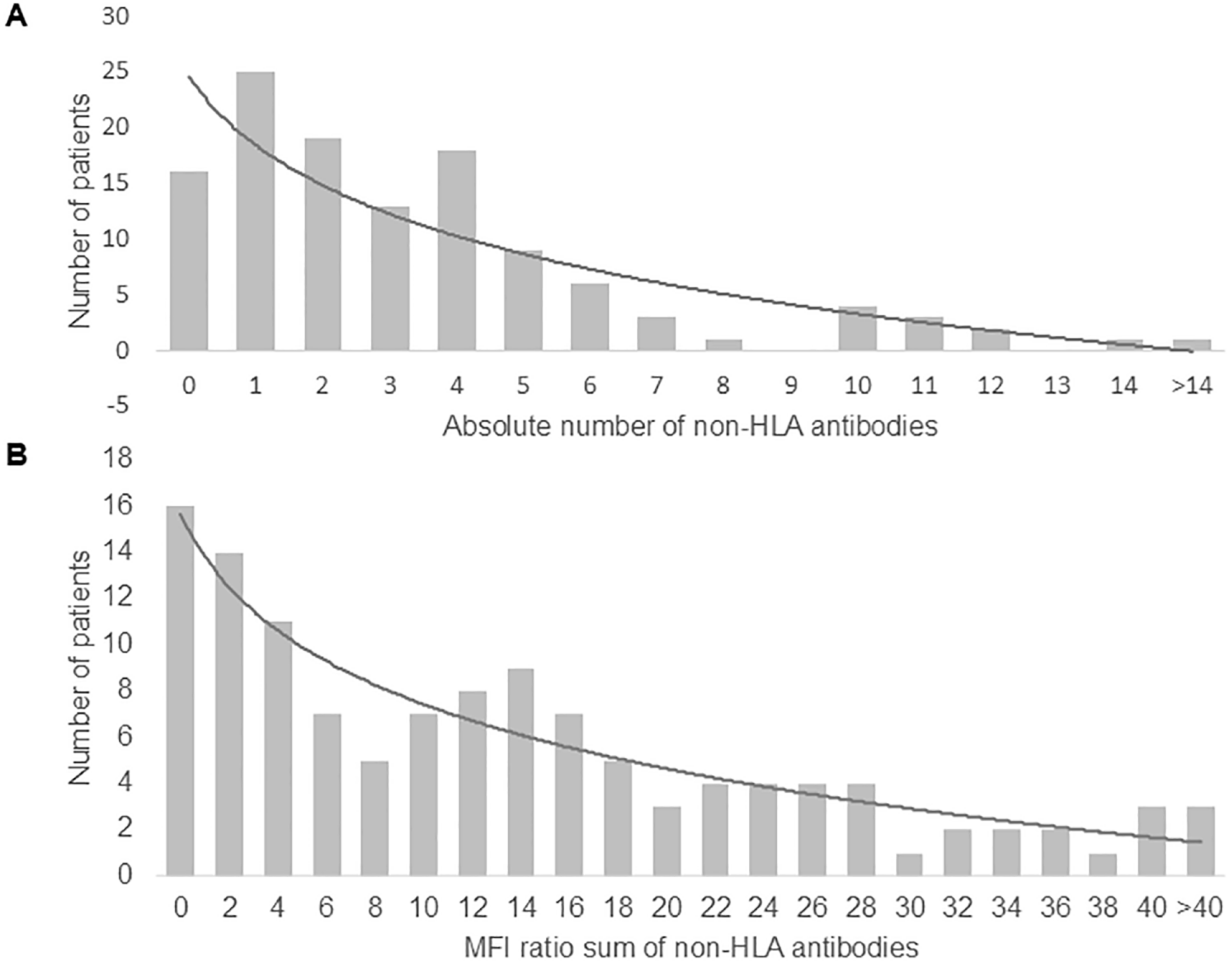
Distribution of patients with pretransplant non-HLA antibodies according to **(A)** the absolute number of non-HLA antibodies and **(B)** the MFI ratios sum of the positive non-HLA antibodies per patient.

Of the 121 patient serum samples analyzed before KT, 105 (87%) patients had one or more non-HLA antibodies according to the manufacturer’s cut-offs, with significantly higher prevalence in AMBR/MVI cases compared to controls (97.8% vs. 80%, p = 0.005). Patients had a median of 3 (IQR 1–4.5) non-HLA antibodies and a median MFI ratio sum of 11.7 (IQR 3.7–21.3). Both showed a logarithmic distribution (**Figure 2**). Compared to controls, patients with ABMR/MVI had a significantly higher number of non-HLA antibodies [median 4 (IQR 2–5) vs. 2 (IQR 1–4), p = 0.001] and a higher MFI ratio sum [median 14.4 (IQR 7.8–26.2) vs. 7.8 (IQR 1.8–16.8), p = 0.001].

To assess the association between non-HLA antibodies and ABMR/MVI risk, logistic regression analyses were performed, including the MFI ratio sum of non-HLA antibodies along with baseline and transplant characteristics. Patients with a higher non-HLA MFI ratio sum exhibited higher odds of developing ABMR/MVI [OR = 1.039, CI (95%) 1.008–1.072, p = 0.014], independently of their HLA sensitization status (HLA cPRA over 50% before KT), previous transplantation, and recipient sex (**Table 2**). This association remained significant after adjusting for the presence of HLA-DSA before KT confirming that the non-HLA antibody effect persists in this context (**Supplementary Table 2A**). Finally, an analysis using the absolute count of positive non-HLA antibodies yielded similar results (**Supplementary Table 2B**).

**Table 2:** Univariable and multivariable logistic regression analysis for ABMR/MVI development according to HLA and non-HLA antibodies before transplantation.

	OR	p-Value	CI (95%)
Univariable			
Recipient sex (female)	2.932	0.006	1.372–6.265
Recipient age (years)	0.993	0.634	0.966–1.021
Body mass index (kg/m^2^)	1.005	0.873	0.942–1.072
Cause of end-stage renal disease (immune mediated)	0.917	0.860	0.352–2.392
Preemptive KT	1.503	0.463	0.506–4.464
Median time on dialysis (months)	1.003	0.703	0.987–1.019
Donor age (years)	0.992	0.565	0.967–1.018
Donor type (live donor)	1.024	0.958	0.420–2.500
Median cold ischemia time (h)	1.016	0.591	0.959–1.076
Previous transplantation	3.186	0.078	0.878–11.563
HLA cPRA over 50% before KT	3.827	0.009	1.396–10.492
Non-HLA MFI ratio sum before KT	1.040	0.010	1.010–1.072
Multivariable			
HLA cPRA over 50% before KT	2.369	0.183	0.666–8.424
Non-HLA MFI ratio sum before KT	1.039	0.014	1.008–1.072
Recipient sex (female)	2.011	0.104	0.867–4.665
Previous transplantation	1.738	0.503	0.345–8.765

**Figure 3:**
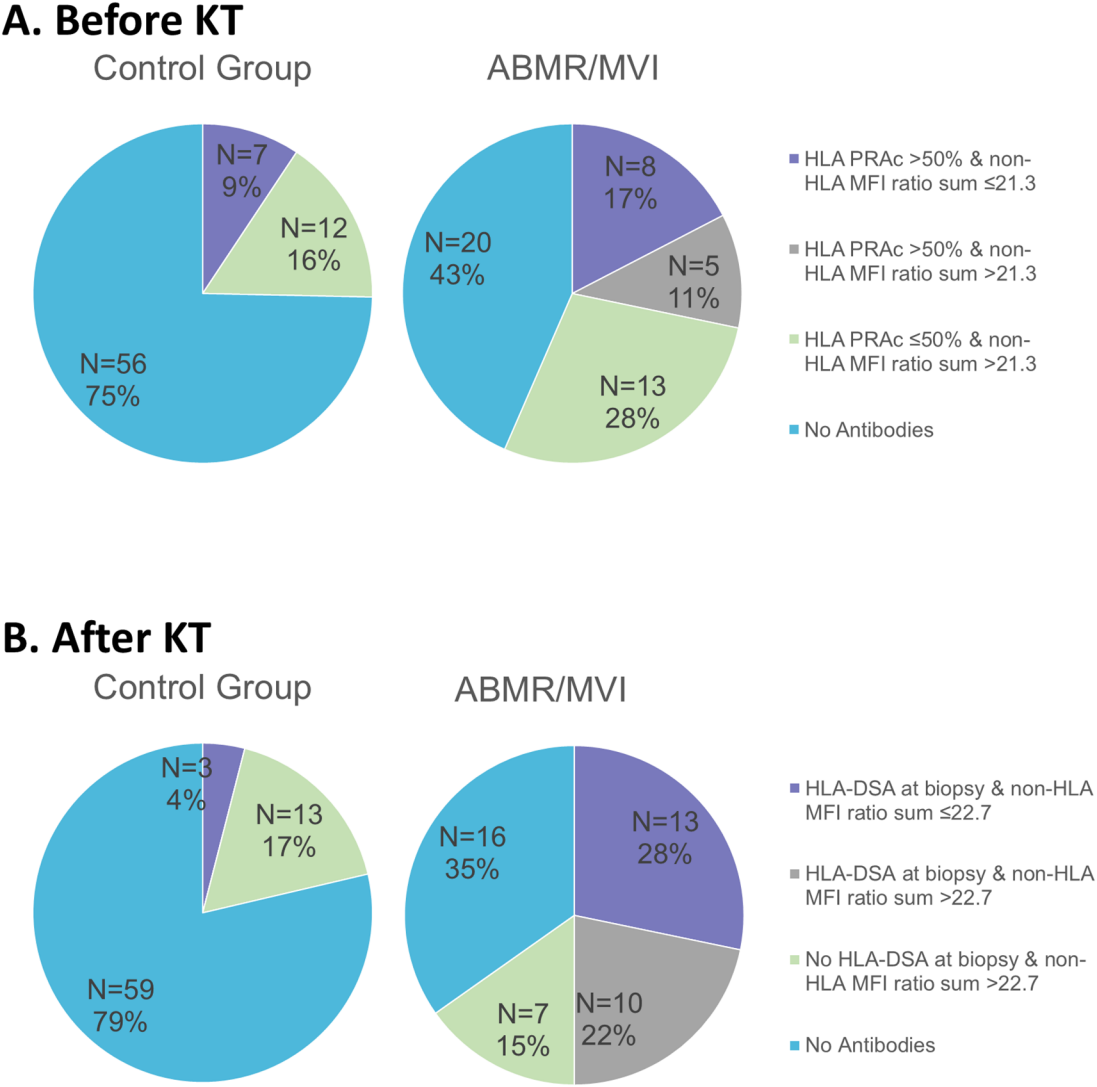
Clinical picture of ABMR/MVI and detection of HLA and non-HLA antibodies: **(A)** Before transplantation, **(B)** After transplantation.

Aiming to elucidate the potential role of multiplex non-HLA tests in the KT recipient assessment, patients were stratified into quartiles based on the non-HLA antibody burden before KT (which combines the number and strength of antibodies). Patients in the fourth quartile of the non-HLA MFI ratio sum (MFI ratio sum >21.3) exhibited the highest odds of developing ABMR/MVI [OR = 5.033, CI (95%) 1.523–16.626, p = 0.008], independently of their HLA sensitization status before KT (**Supplementary Table 2C**). Among the patients with ABMR/MVI, 39.1% (n = 18) exhibited a non-HLA antibody MFI ratio sum >21.3 before KT (vs. 16% of the controls, p = 0.004) and 28.3% (n = 13) had an HLA cPRA over 50% (vs. 9.3% of the controls, p = 0.007) (**Figure 3A**). Among the cases with ABMR/MVI lacking detectable HLA and non-HLA antibodies before KT (n = 20), seven presented with *de novo* HLA-DSA at the time of biopsy diagnosis.

We also analyzed whether the cellular localization of the non-HLA antigens could play a role in how the antibodies targeting them associate with the development of ABMR/MVI. The 60 non-HLA panel antigens were classified into extracellular (secreted or plasma membrane) and intracellular antigens (**Supplementary Table 1**). Logistic regression analyses showed that, before KT, the MFI ratio sum of extracellular, but not intracellular, non-HLA antigens was independently associated with higher odds of ABMR/MVI [OR = 1.053, CI (95%) 1.002–1.107, p = 0.040] (**Table 3**).

**Table 3:** Univariable and multivariable logistic regression analysis for ABMR/MVI development according to non-HLA antigen localization.

	OR	p-Value	CI (95%)
Univariable			
Recipient sex (female)	2.932	0.006	1.372–6.265
Recipient age (years)	0.993	0.634	0.966–1.021
Body mass index (kg/m^2^)	1.005	0.873	0.942–1.072
Cause of end-stage renal disease (immune mediated)	0.917	0.860	0.352–2.392
Preemptive KT	1.503	0.463	0.506–4.464
Median time on dialysis (months)	1.003	0.703	0.987–1.019
Donor age (years)	0.992	0.565	0.967–1.018
Donor type (live donor)	1.024	0.958	0.420–2.500
Median cold ischemia time (h)	1.016	0.591	0.959–1.076
Previous transplantation	3.186	0.078	0.878–11.563
HLA cPRA over 50% before KT	3.827	0.009	1.396–10.492
Extracellular non-HLA MFI ratio sum before KT	1.051	0.035	1.003–1.100
Intracellular non-HLA MFI ratio sum before KT	1.045	0.048	1.000–1.092
A. Multivariable, non-HLA as extracellular MFI ratio sum			
HLA cPRA over 50% before KT	2.701	0.129	0.748–9.756
Extracellular non-HLA MFI ratio sum before KT	1.053	0.040	1.002–1.107
Recipient sex (female)	1.936	0.123	0.836–4.480
Previous transplantation	1.606	0.561	0.325–7.932
B. Multivariable, non-HLA as intracellular MFI ratio sum			
HLA cPRA over 50% before KT	2.110	0.245	0.599–7.440
Intracellular non-HLA MFI ratio sum before KT	1.044	0.069	0.997–1.093
Recipient sex (female)	2.296	0.048	1.006–5.238
Previous transplantation	1.607	0.563	0.322–8.011

Last, we assessed the non-HLA impact on clinical outcomes. Patients in the fourth quartile of the non-HLA MFI ratio sum (>21.3) exhibited significantly worse graft function and proteinuria at biopsy time (**Supplementary Table 3A**). However, these associations were not significant after adjusting for ABMR/MVI diagnosis in a regression analysis. On graft survival, we found no association with high non-HLA ratio sum. In a Cox regression analysis, only ABMR/MVI diagnosis was significantly associated with graft loss [OR = 6.577, CI (95%) 1.830–23.631, p = 0.004] (**Supplementary Table 3B**).

When evaluating individual non-HLA antibodies before KT, 6 out of the 60 antibodies analyzed were significantly associated with ABMR/MVI in a univariable logistic regression analysis (p < 0.1, **Supplementary Table 4**). In a multivariable analysis including these six antibodies, along with the presence of an HLA cPRA over 50% before KT and clinical characteristics, we identified that only the detection of antibodies against GSTT1 remained independently associated with ABMR/MVI [OR = 3.664, CI (95%) 1.234–10.883, p = 0.019] (**Table 4**).

**Table 4:** Multivariable logistic regression analysis of pretransplant non-HLA antibodies significantly associated with ABMR/MVI and HLA sensitization.

	OR	p-Value	CI (95%)
APOL2	4.820	0.073	0.862–26.949
EMCN	2.358	0.260	0.530–10.504
GSTT1	3.664	0.019	1.234–10.883
Nucleolin	10.032	0.061	0.899–111.922
PLA2R1	11.040	0.061	0.894–136.391
Thyroglobulin	1.978	0.196	0.703–5.563
HLA cPRA over 50% before KT	2.393	0.253	0.537–10.668
Recipient sex (female)	2.533	0.073	0.916–7.008
Previous transplantation	1.925	0.460	0.338–10.959

### Non-HLA antibodies after transplantation

At the time of biopsy, 103 of 121 patients (85.1%) had one or more non-HLA antibodies, similar to the proportion observed before KT (87%). Specifically, 96 (79%) of the KT recipients maintained the presence of non-HLA antibodies, 9 (7.4%) lost them, and 7 (5.8%) developed *de novo* non-HLA antibodies. There were no significant differences in the number of antibodies before and after KT (median 3, IQR 1–5; p = 0.722, Wilcoxon test), the MFI ratio sum [median before KT 11.7 (IQR 3.7–21.3) vs. after KT 11.6 (IQR 4.5–22.7); p = 0.767, Wilcoxon test], or its distribution (**Supplementary Figure**). Patients who developed *de novo* non-HLA antibodies after transplantation (n = 7) presented a lower non-HLA antibody number [median 2 (IQR 1–8) vs. 3 (IQR 2–6), p = 0.555] and MFI ratio sum [median 6.7 (IQR 4.6–29.8) vs. 14.37, IQR (6.9–23.1), p = 0.283] compared with persisting non-HLA antibodies, though not reaching statistical significance. Histological characteristics of biopsies are detailed in **Supplementary Table 5**.

Similar to the findings before KT, a higher non-HLA MFI ratio sum at biopsy time was associated with increased odds of ABMR/MVI [OR = 1.031, CI (95%) 1.002–1.060, p = 0.035] independent of HLA-DSA and recipient sex (**Table 5A**), although no association was found with absolute antibody count (data not shown). Among cases with ABMR/MVI, 37% (n = 17) exhibited a non-HLA antibody MFI ratio sum >22.7 at biopsy (vs. 17% of controls, p = 0.015), and 22% (n = 10) also had HLA-DSA. Of the patients, 35% had no HLA-DSA nor high non-HLA burden (vs. 79% of controls, p < 0.001) (**Figure 3B**). When replicating the extracellular and intracellular non-HLA antibodies analysis at biopsy time, only antibodies against intracellular non-HLA antigens were significantly associated with ABMR/MVI [OR = 1.062, CI (95%) 1.011–1.116, p = 0.018) (**Tables 5B, C**).

**Table 5:** Univariable and multivariable logistic regression analysis for ABMR/MVI development according to HLA and non-HLA antibodies after KT, at biopsy time.

	OR	p-Value	CI (95%)
Univariable			
Recipient sex (female)	2.932	0.006	1.372–6.265
Recipient age (years)	0.993	0.634	0.966–1.021
Body mass index (kg/m^2^)	1.005	0.873	0.942–1.072
Cause of end-stage renal disease (immune mediated)	0.917	0.860	0.352–2.392
Preemptive KT	1.503	0.463	0.506–4.464
Median time on dialysis (months)	1.003	0.703	0.987–1.019
Donor age (years)	0.992	0.565	0.967–1.018
Donor type (live donor)	1.024	0.958	0.420–2.500
Median cold ischemia time (h)	1.016	0.591	0.959–1.076
HLA-DSA after KT	24.000	<0.001	6.597–87.315
Non-HLA MFI ratio after KT	1.039	0.006	1.011–1.067
Extracellular non-HLA MFI ratio sum after KT	1.060	0.015	1.011–1.111
Intracellular non-HLA MFI ratio sum after KT	1.075	0.002	1.027–1.126
A. Multivariable, non-HLA as MFI ratio sum			
HLA-DSA after KT	24.752	<0.001	6.352–96.355
Non-HLA MFI ratio after KT	1.031	0.035	1.002–1.060
Recipient sex (female)	3.399	0.011	1.317–8.769
B. Multivariable, non-HLA as extracellular MFI ratio sum			
HLA-DSA after KT	24.984	<0.001	6.462–96.599
Extracellular non-HLA MFI ratio sum after KT	1.050	0.062	0.998–1.106
Recipient sex (female)	3.392	0.011	1.326–8.675
C. Multivariable, non-HLA as intracellular MFI ratio sum			
HLA-DSA after KT	25.027	<0.001	6.426–97.631
Intracellular non-HLA MFI ratio sum after KT	1.062	0.018	1.011–1.116
Recipient sex (female)	3.074	0.021	1.186–7.964

Finally, we individually assessed the association of each non-HLA antibody in serum after KT. In a multivariable analysis, including only the individual antibodies significantly (p < 0.01) associated with ABMR/MVI in previous univariable analysis, only GSTT1 was associated with ABMR/MVI at the time of biopsy [OR = 4.887, CI (95%) 1.740–13.729, p = 0.003] consistent with pretransplant findings (**Supplementary Table 6**).

## Discussion

ABMR stands as a significant contributor to graft loss after KT, with potential development even in the absence of detectable HLA-DSA. Several studies have estimated its prevalence to range from 30% to 60% among biopsies showing histological features suggestive of ABMR (13, 16, 32). Various investigations on non-HLA antibodies have unveiled their potential involvement in ABMR. These studies have assessed the impact of individual antibodies targeting specific molecules, and more recently, they have delved into the analysis involving antibodies against a broader spectrum of molecules (27, 28, 30).

In our study, we examined multiple non-HLA targets and observed that a high overall burden of non-HLA antibodies, both before and after KT, correlated with antibody-mediated damage, encompassing both ABMR and MVI, regardless of the detection of HLA antibodies. Specifically, antibodies targeting extracellular antigens seem to have a predominant role before KT, while those targeting intracellular antigens do it at diagnosis. We postulate that incorporating non-HLA antibodies into the pretransplant immunological risk assessment could prove beneficial.

Using a multiplex test comprising 60 non-HLA antibodies and the manufacturer’s recommended cut-off, we observed that non-HLA antibodies are frequently present in our patients. Specifically, 87% of the recipients exhibited at least one non-HLA antibody before KT. This finding aligns with previous reports: employing a 62-antigen multiplex test, Delville et al. (27) detected non-HLA antibodies in 83% of the patients, and Senev et al. (28) found that 98.7% of the patients had at least one non-HLA antibody before KT when employing an 82-antigen multiplex test. The high prevalence in our study, combined with the fact that before KT, surface and secreted non-HLA antigens seem to be primarily associated with ABMR/MVI, aligns with an autoimmune origin of these antibodies targeting self-molecules arising during the advanced stages of kidney disease as previously described (28, 33).

Our data showed that patients harboring a high pretransplant non-HLA antibody strength, particularly against extracellular targets, had an increased risk of ABMR/MVI. This association persisted after KT. While the association between non-HLA antibodies and ABMR has been explored previously, prior studies have focused on specific patient subsets without HLA-DSA (28) and many assessed sera only at the time of ABMR diagnosis (27, 30, 33). In an analysis before KT integrating multiple tests, Delvile et al. (27) concluded that before KT, non-HLA antigens expressed in the glomerular endothelia, in the absence of HLA-DSA, were linked to early ABMR in a highly selective cohort. In addition, Senev et al. (28) reported that a high non-HLA antibody burden before KT presented an increased risk of developing ABMR in the absence of HLA-DSA. In contrast, our cohort included patients with and without HLA-DSA, and we incorporated an evaluation setting before KT, including HLA sensitization (cPRA), in our analysis. Our findings indicate a significant association between a high non-HLA antibody MFI ratio sum and an augmented risk of ABMR/MVI in our cohort independent of HLA-DSA as a risk factor.

A non-HLA antibody evaluation test could help clinicians more accurately identify recipients at increased risk of developing ABMR/MVI. We propose the integration of non-HLA antibodies into the existing antibody evaluations, analogous to how cPRA is used to estimate a recipient’s HLA sensitization status before a donor is selected, which has been associated with rejection and graft survival (34). However, we suggest that a more restrictive cut-off value for these tests is needed to improve specificity and reduce false positivities. We have used the quartile division to identify patients at risk (MFI ratio sum >21.3 before KT and >22.7 after KT), but further research is required to define a validated threshold to get a more effective biomarker. Moreover, it is also necessary to validate whether only those non-HLA antibodies against extracellular, but not intracellular, antigens are relevant to assess risk before KT.

Even with the incorporation of non-HLA antibody evaluation before KT, 16 out of the 46 patients who developed MVI or ABMR showed neither a high non-HLA MFI ratio sum nor detectable HLA-DSA. Consequently, while the non-HLA antibodies before KT may aid in identifying patients with an increased ABMR/MVI risk independent of HLA sensitization, they are either an imperfect marker or not solely responsible for a proportion of these cases.

Among the 60 individual antibodies assessed, only antibodies against GSTT1 were individually associated with ABMR/MVI, both before and after KT. GSTT1 is a cytosolic protein involved in oxidative cellular damage. Approximately 20% of the Caucasian population lacks this protein due to gene deletion. Aguilera et al. (35–37) linked a donor–recipient genetic incompatibility to hepatitis in liver transplantation (36) and demonstrated that the development of GSTT1 antibodies after KT was associated with ABMR and C4d deposits (37, 38). Comoli et al. (23) recently described an association between GSST1 antibodies and ABMR in a pediatric KT cohort. Our results support these associations and extend them to the broader context of ABMR/MVI before and after KT.

Individual antigens identified to be significantly associated with ABMR risk often differ between studies (26–28, 39). This could be caused by the differences in cohorts, the design of the kits used, or the limited individual effect of each antibody. Based on our findings and prior evidence, we consider that the total number of non-HLA antibodies targeting different antibodies and the concentration of these antibodies before KT may have a greater effect on the risk of developing ABMR than the presence of antibodies to various antigens separately. This is consistent with previous findings from our group regarding other individual non-HLA antigens such as AT1R, endothelin receptor, and MICA (16).

After transplantation, we also found that a high non-HLA antibody burden is associated with an ABMR/MVI diagnosis. Interestingly, in our experience, only the MFI ratio sum, and not the absolute number, of non-HLA antibodies significantly correlated with the occurrence of ABMR/MVI. This observation supports the relevance of the antibody strength as a surrogate of its relative amount in circulation. Nevertheless, it is also relevant to acknowledge that antibodies in our study were considered positive with an MFI ratio ≥1, as indicated by the manufacturer, which could be seen as a lax cutoff that may result in false-positive antibodies when considering only the absolute count and not the MFI.

Notably, the cell localization of the target of those non-HLA antibodies appears to shift after transplantation. A high burden of non-HLA antibodies against intracellular antigens, but not extracellular ones, was associated with ABMR/MVI at diagnosis. As some authors have previously postulated, graft damage occurring during or after transplantation may result in the exposure of intracellular antigens and trigger the development of these antibodies (40).

We consider our study clinically relevant, as it suggests the potential utility of detecting non-HLA antibodies against extracellular targets in the recipients’ immunological risk assessment before transplantation. This additional evaluation may enhance clinicians’ ability to identify individuals at heightened risk of ABMR/MVI, thereby allowing for more tailored management strategies and potentially improving transplant outcomes.

Our study has some limitations. First, it is a single-center, retrospective observational study, which limits the generalizability of the findings without an external validation cohort. Second, the cohort is heterogeneous, encompassing patients with full ABMR and subclinical MVI, mixing indication and protocol biopsies. However, previous studies have already focused on highly selected cases (27, 28), and our findings were consistent even when including more diverse controls and MVI and ABMR cases. Third, our multiplex panel included a limited selection of non-HLA antigens and excluded classical ones such as anti-AT1R. Consequently, antibodies against these antigens were not assessed in our cohort, and their potential synergistic role in ABMR could not be explored. However, other studies using similar multiplex tests found no association between anti-AT1R and ABMR (28,30).

In conclusion, our study demonstrated that non-HLA antibodies are detectable in approximately 87% of KT recipients both before and after transplantation using a non-HLA single antibody multiplex test and the manufacturer’s recommended cut-off. Importantly, we found that a high non-HLA antibody burden before KT, particularly those antibodies targeting extracellular antigens, was independently associated with an increased risk of ABMR/MVI, even after accounting for overall HLA sensitization and the presence of HLA-DSA. These findings support the potential value of incorporating non-HLA evaluation into the current pretransplant immunological risk assessment, solely focused on HLA sensitization. Further prospective studies are required to validate these observations and to optimize cut-off values. Ultimately, this may enable a clinical implementation of non-HLA antibody testing that could improve KT recipients’ risk stratification and management strategies in KT.

## Data Availability

The raw data supporting the conclusions of this article will be made available by the authors, without undue reservation.
