# Supplementary material for: Risk assessment of antibody-mediated damage based on the detection of HLA and non-HLA antibodies toward extracellular antigens before kidney transplantation
Source: Front Immunol. 2025 Aug 12;16:1614408. doi: 10.3389/fimmu.2025.1614408 (PMC12378050; doi:10.3389/fimmu.2025.1614408)
Supplement: Supplementary file 1 [file DataSheet1.pdf]

SUPPLEMENTARY MATERIAL

Supplement Table 1. Antigen list of Immucor® prototype assay for detection of 60 non-HLA antibodies.

Probe	Antigen	MFI Cutoff	MFI pre-KT (median, IQR)		Description	Localization
			No ABMR/MVI	ABMR/MVI		
**1**	Actin	278	115.8 (80.3-175.8)	147 (89.3-226)	Actin	Intracellular
**2**	AGRN	1168	342 (264.1-566.5)	409 (278-619.5)	Agrin	Plasma membrane
**3**	APOL2	420	174 (112-244.6)	177 (137-308.5)	Apolipoprotein L, 2	Intracellular
**4**	ARHGDIB	1364	206.8 (136.6-330.8)	237.5 (131-443.3)	Rho GDP Dissociation Inhibitor Beta	Intracellular
**5**	ATP5B	925	230.8 (149.3-393.3)	250 (184.8-433)	ATP synthase, H+ transporting, mitochondrial F1 complex, beta polypeptide	Intracellular
**6**	CCP	168	97 (75-141.5)	153.8 (105.4-224.8)	Cyclic citrullinated peptide	Secreted
**7**	CD40	417	153.8 (105.4-224.8)	170 (117.5-236.5)	CD40 molecule, TNF receptor superfamily member 5	Plasma membrane / Secreted
**8**	CGB5	794	142.5 (215.3-334.8)	248 (149.3-316.5)	Chorionic gonadotropin, beta polypeptide 5	Secreted
**9**	Collagen I	409	180.5 (134.4-249.3)	165 (148-221.8)	Collagen I	Secreted
**10**	Collagen II	740	344.3 (247.5-500.6)	340 (232-673.5)	Collagen II	Secreted
**11**	Collagen III	1856	711 (469.6-1088.8)	643 (452.3-902.5)	Collagen III	Secreted
**12**	Collagen IV	478	208 (167.8-302.1)	215 (167.2)	Collagen IV	Secreted
**13**	Collagen V	724	339,2 (239.9-544.6)	273 (216-521.3)	Collagen V	Secreted

**14**	Collagen VI	1164	499 (334.3-730.5)	482 (327.8-749.5)	Collagen VI	Secreted
**15**	CSF2	313	135,5 (86.4-203.6)	148 (95-244.5)	Colony stimulating factor 2	Secreted
**16**	CXCL11	455	188,8 (128,3-271)	182 (132.8-270.3)	Chemokine (C-X- C motif) ligand 11	Secreted
**17**	CXCL9	368	149,3 (101.1-212.8)	175 (118.5-221.5)	Chemokine (C-X-C Motif) 9	Secreted
**18**	DEXI	929	299,3 (188-455,8)	304 (233-522,5)	Dexamethasone-induced transcript	Plasma membrane
**19**	EMCN	355	138,3 (84,3-251)	169 (108,8-271,3)	Endomucin	Intracellular
**20**	ENO1	2168	560.3 (261.8-1691.4)	596 (299,3-2049)	Alpha-enolase	Intracellular
**21**	FAS	214	102.3 (68.6-138.9)	116 (87-163,5)	Fas cell surface death receptor	Plasma membrane /Secreted
**22**	FLRT2	1569	671,5 (429,25-960,13)	662 (449,25-1134,75)	Leucine-rich repeat transmembrane protein FLRT2	Plasma membrane /Secreted
**23**	FN1	673	186,5 (113,13-274,75)	232 (153,5-341,75)	Fibronectin 1	Secreted
**24**	GAPDH	662	162,5 (102,5-264,63)	267 (138-398)	Glyceraldehyde- 3-phosphate dehydrogenase	Plasma membrane
**25**	GDNF	481	154,5 (101-274,75)	178,5 (122-280)	Glial cell-derived neurotrophic factor	Secreted
**26**	GSTT1	588	223,25 (138,5-402,5)	296 (171,5-1017,5)	Glutathione S- Transferase theta-1	Intracellular
**27**	HARS	1326	302,5 (210,75-560,5)	360 (242-638,5)	Histidyl-tRNA, Jo-1	Intracellular
**28**	HSPB1	362	138,25 (94,25-203,13)	182 (126,75-272,5)	Heat shock protein beta-1	Intracellular
**29**	ICAM1	235	91,5 (62,25-125,75)	111 (85-148)	Intracellular Adhesion Molecule 1	Plasma membrane
**30**	IFNG	332	120 (89-185)	158 (114,75-235,5)	Interferon Gamma	Secreted

**31**	IL-21	325	138,5 (92,88-214,13)	187 (125-271,75)	Interleukin 21	Secreted
**32**	IL-8	952	285,75 (173,1-501,7)	333 (201,5-528,25)	Interleukin 8, CXCL8	Secreted
**33**	KRT18	1348	335,25 (246,38-521)	423 (265,5-877)	Cytokeratin 18	Intracellular
**34**	KRT8	982	304 (203,38-406,38)	345 (253-490,25)	Cytokeratin 8	Intracellular
**35**	LGALS3	488	151,5 (105-229,88)	183 (119,5-322,5)	Lectin, galactoside- binding, soluble, 3	Intracellular
**36**	LGALS8	779	273,25 (182,1-434,59	350 8 (201,5-524,5)	Lectin, galactoside- binding, soluble, 8	Intracellular
**37**	LMNA	1994	417,25 (259,75-843,6)	656 (273,5-988,5)	Prelamin-A/C	Intracellular
**38**	LPHN1	1149	230,75 (161,25-350,5)	315 (213,5-501,5)	Latrophilin 1	Plasma membrane
**39**	Myosin	5457	2191, (1197,5-3342,1)	1728 (1122,5-3617)	Myosin, Human	Intracellular
**40**	NCL	1622	402,5 (207-684,25)	371,5 (242,5-644,5)	Nucleolin	Intracellular
**41**	P2RY11	443	171,25 (119,5-268,25)	221 (149-327,75)	Purinergic receptor P2Y, G- protein-coupled, 11	Plasma membrane
**42**	PECR	1244	357 (215,38-751,88)	363,5 (254,3-645,8)	Peroxisomal trans-2-enoyl- CoA Reductase	Intracellular
**43**	PLA2R1	698	270,25 (191,1-426,25)	340 (234,5-501)	Phospholipase A2 receptor 1, 180kDa	Plasma membrane /Secreted
**44**	PRKCH	1580	395 (240,63-741,5)	454 (290,5-969,5)	Protein kinase C, eta	Intracellular
**45**	PRKCZ	1928	533 (296-3367,5)	619 (361,50-2386)	Protein kinase C, zeta	Intracellular
**46**	PTPRO	515	187,75 (141-271,5)	211 (149,5-328,25)	Receptor-type Tyrosine-protein Phosphatase U	Plasma membrane
**47**	ROR1	405	143 (100,25-208,75)	168 (116-282)	Receptor Tyrosine Kinase- Like Orphan Receptor 1	Plasma membrane

**48**	SHC3	1526	389,25 (262,63-652)	388 (260,75-645,5)	SHC Adaptor Protein 3	Plasma membrane
**49**	SNRPB2	2140	405,5 (228,75-757,88)	492 (272-869,5)	Small nuclear ribonucleoprotein polypeptide B	Intracellular
**50**	SNRPN	2183	481 (290,88-941,25)	595 (387,25-1131)	Small Nuclear Ribonucleoprotein Polypeptide N	Intracellular
**51**	SSB	2909	453 (263,75-1297)	673,5 (325,75-1506)	Sjogren syndrome antigen B (autoantigen La)	Intracellular
**52**	STAT6	633	223 (147,25-410,5)	225 (161-426,50)	Signal Transducer and Activator of Transcription 6	Intracellular
**53**	TG	1341	652,25 (321,13-1090,25)	922 (383,5-2560,25)	Thyroglobulin	Secreted
**54**	Transferrin	592	188 (149,13-261,75)	213 (166,75-278,75)	Transferrin (negative control)	Plasma membrane /Secreted
**55**	TUBA1B	402	155,25 (101,88-213,25)	180 (119,75-270)	Tubulin, alpha 1b	Intracellular
**56**	TUBB	695	212,75 (151-299,38)	237,5 (170-347,25)	Tubulin beta	Intracellular
**57**	Tubulin	413	146 (96,5-202,38)	163 (111-273,5)	Tubulin	Intracellular
**58**	VCL	8103	1322,5 (707,88-3027,38)	2094 (872-2963,5)	Vinculin	Plasma membrane
**59**	VEGFA	810	220 (149,75-306,88)	211 (158,5-306)	Vascular endothelial growth factor-A	Secreted
**60**	VIM	406	154,25 (99,63-208,88)	190 (128,25-262)	Vimentin	Plasma membrane

Supplement Table 2. Univariable and Multivariable logistic regression analysis for the ABMR/MVI development according to HLA-DSA and non-HLA antibodies.

	OR	p=value	CI (95%)
**Univariable**			
Recipient sex (female)	2.932	0.006	1.372-6.265
Recipient age (years)	0.993	0.634	0.966-1.021
Body mass index (Kg/m2)	1.005	0.873	0.942-1.072
Cause of end-stage renal disease (Immune-mediated)	0.917	0.860	0.352-2.392
Preemptive KT	1.503	0.463	0.506-4.464
Median time on dialysis (months)	1.003	0.703	0.987-1.019
Donor age (years)	0.992	0.565	0.967-1.018
Donor type (live donor)	1.024	0.958	0.420-2.500
Median cold ischemia time (hours)	1.016	0.591	0.959-1.076
Previous transplantation	3.186	0.078	0.878-11.563
HLA cPRA over 50% before KT	3.827	0.009	1.396-10.492
HLA-DSA before KT	8.878	0.007	1.824-43.208
Non-HLA MFI ratio sum before KT	1.040	0.010	1.010-1.072
Absolute non-HLA count before KT	1.121	0.121	1.001-1.254
**A. Multivariable, non-HLA as MFI ratio sum**			
HLA-DSA before KT	9.034	0.068	0.848-96.191
Non-HLA MFI ratio sum before KT	1.036	0.024	1.005-1.068
Recipient sex (female)	2.327	0.043	1.028- 5.270
Previous transplantation	0.742	0.815	0.061-9.048
**B. Multivariable, non-HLA as an absolute count**			
HLA-DSA before KT	8.993	0.071	0.831-97.282
Absolute non-HLA count before KT	1.122	0.049	1.000-1.258
Recipient sex (female)	2.624	0.019	1.169-5.893
Previous transplantation	0.744	0.816	0.062-8.943
**C. Multivariable, non-HLA as MFI ratio sum quartiles**			
HLA cPRA over 50% before KT	2.200	0.216	0.631-7.670
Non-HLA MFI ratio sum (ref. Q1) before KT			
- Q2 non-HLA MFI ratio sum	1.234	0.741	0.354-4.301
- Q3 non-HLA MFI ratio sum	2.120	0.213	0.650-6.916
- Q4 non-HLA MFI ratio sum	5.033	0.008	1.523-16.626
Recipient sex (female)	2.025	0.107	0.858-4.779
Previous transplantation	2.372	0.322	0.430-13.095

Supplement Table 3A. Clinical outcomes according to non-HLA MFI ratio sum (MFI ratio sum above 21.3).

	Low non-HLA MFI ratio sum (n=91)	High non-HLA MFI ratio sum (n=30)	p=value
Graft loss death censored at end of follow-up, n (%)	7 (7.7)	8 (26.7)	0.006
Renal function:			
- Serum creatinine (mg/dl) mean ± SD	1.52 ± 0.71	1.8± 0.64	0.056
- eGFR (MDRD) (ml/min) mean ± SD	41.97 ± 17.12	51.80 ± 51.81	0.021
- Urine protein/creatinine ratio (mg/g) mean ± SD	272.75 ± 358.211	527.85 ± 660.25	0.008

Supplement Table 3B. Univariable Cox regression analysis for graft loss risk, death censored, non-HLA as MFI ratio sum

	HR	p=value	CI (95%)
Recipient sex (female)	1.081	0.889	0.363-3.220
Recipient age (years)	1.016	0.445	0.976-1.058
Cause of end-stage renal (Immune-mediated disease)	1.124	0.857	0.315-4.016
Preemptive KT	1.345	0.699	0.300-6.033
Donor age (years)	1.028	0.171	0.988-1.069
Donor type (live donor)	0.662	0.588	0.149-2.945
Median cold ischemia time (hours)	1.036	0.403	0.954-1.124
HLA cPRA over 50% before KT	1.058	0.932	0.292-3.829
ABMV/MVI diagnosis during follow-up	6.577	0.004	1.830-23.631
Non-HLA MFI ratio sum before KT	1.020	0.149	0.993-1.048

Supplement Table 4. Univariable logistic regression analysis for pretransplant non-HLA antibodies significantly associated with ABMR/MVI

	N	OR	p=value	CI (95%)
APOL2	10	4.308	0.042	1.054-17.600
EMCN	13	3.196	0.074	0.901-9.641
GSTT1	23	3.208	0.015	1.256-8.195
Nucleolin	6	9.024	0.048	1.019-79.887
PLA2R1	5	7.048	0.085	0.763-65.136
Thyroglobulin	29	2.108	0.084	0.904-4.918

Supplement Table 5. Histological characteristics of biopsies according to HLA and non-HLA antibodies.

	**HLA-DSA before KT or *de novo***	**Non-HLA MFI ratio sum ≥21.3 before KT**	**Both (HLA-DSA & non-HLA ≥21.3)**
N=	16	18	11
Glomerulitis (g) score>0, n(%)	9 (56.2%)	8 (44.4%)	10 (90.9)
Peritubular capilaritis (ptc) score>0, n(%)	12 (75)	5 (27.8)	10 (90.9)
Endarteritis (v) score>0, n(%)	0 (0)	1 (6)	0 (0)
c4d score >0, n(%)	6 (37.5)	3 (17.6)	3 (27.3)
Interstitial inflammation (i) score >0, n(%)	0 (0)	0 (0)	3 (27.3)
Tubulitis (t) score>0, n(%)	2 (12.5)	0 (0)	5 (45.5)
Chronic allograft glomerulopathy (cg) score>0, n(%)	6 (40)	5 (27.8)	1 (9.1)
Arteriolar hialinosis (ah) score>1, n(%)	3 (18.8)	4 (22.2)	5 (45.5)
Interstitial fibrosis (ci) score>1, n(%)	4 (26.7)	6 (33.3)	2 (18.2)
Tubular atrophy (ct) score>1, n(%)	5 (31.2)	7 (38.9)	3 (27.3)

Supplement Table 6. Regression analysis of non-HLA antibodies at biopsy time significantly associated with ABMR/MVI.

	**OR**	**p=value**	**CI (95%)**	**OR**	**p=value**	**CI (95%)**
	**Univariable**			**Multivariable**		
APOL2	3.600	0.081	0.854-15.176	2.119	0.404	0.363-12.380
EMCN	2.947	0.074	0.901-9.641	2.718	0.158	0.677-10.910
GSTT1	5.384	<0.001	2.098-13.816	4.887	0.003	1.740-13.729
HARS	7.048	0.085	0.763-65.136	4.028	0.300	0.288-56.312
LGALS8	4.451	0.082	0.826-23.975	2.797	0.288	0.419-18.664
LPHN1	4.451	0.082	0.826-23.975	5.358	0.079	0.822-34.911
Nucleolin	4.451	0.082	0.826-23.975	2.426	0.397	0.312-18.881
Thyroglobulin	2.305	0.092	0.872-6.090	1.930	0.248	0.632-5.893
TUBA1B	9.024	0.048	1.019-79.887	2.267	0.523	0.184-27.900

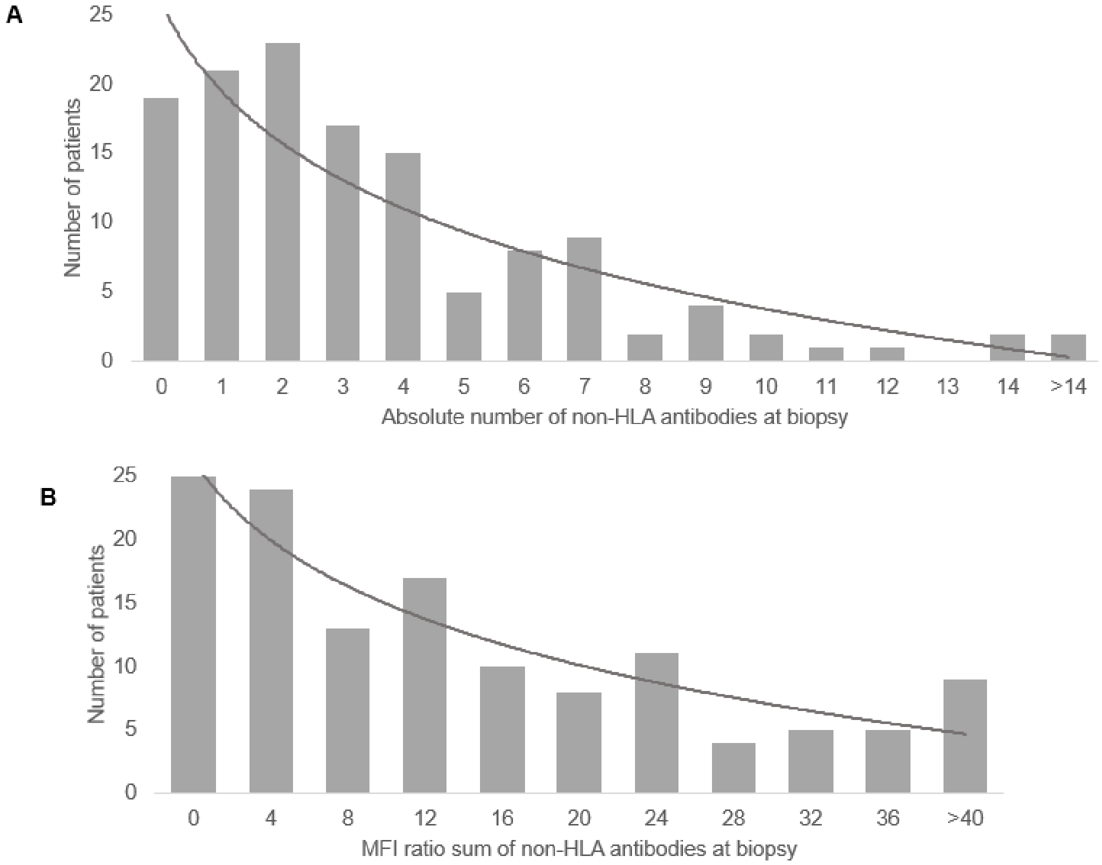

Supplement Figure. Distribution of patients with non-HLA antibodies according to A) absolute number of non-HLA antibodies at biopsy time, B) MFI ratio sum of the positive non-HLA antibodies at biopsy time.
